# Supplementary figures and images for: Effects of tDCS on the attentional blink revisited: A statistical evaluation of a replication attempt
Source: PLoS One. 2022 Jan 27;17(1):e0262718. doi: 10.1371/journal.pone.0262718 (PMC8794161; doi:10.1371/journal.pone.0262718)

anodal cathodal

number of sessions reported

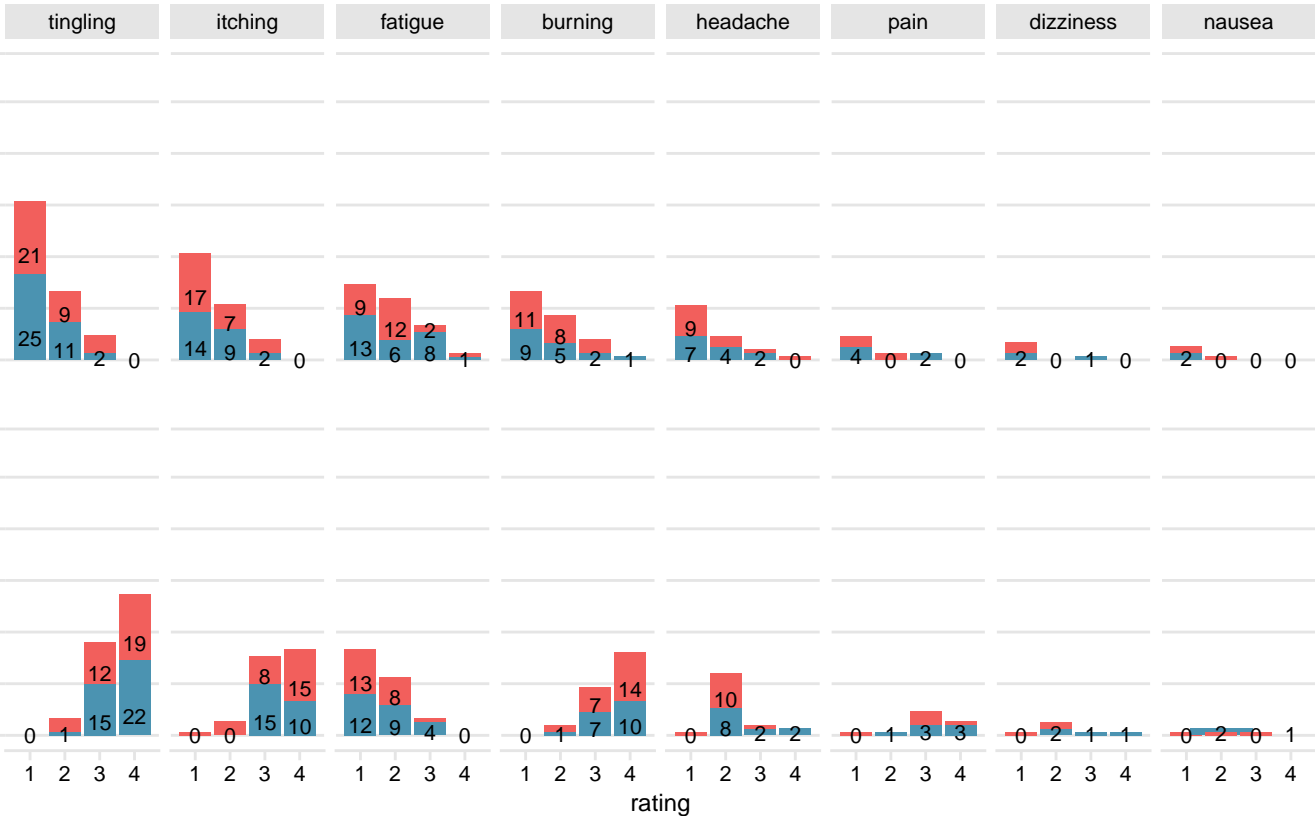

intensity

confidence

rating

Supplement: S1 Fig — Number of reports out of 89 sessions (either anodal or cathodal tDCS). Top row shows intensity ratings [little, moderate, strong, very strong]; bottom row shows participant’s confidence that event was related to tDCS [unlikely, possibly, likely, very likely]. Adverse events are sorted in descending order of number of reports (for very rare events (five reports or fewer for a given polarity), some text counts have been removed to prevent overlap). (PDF) [file pone.0262718.s001.pdf]
