# Supplementary material for: Effects of tDCS on the attentional blink revisited: A statistical evaluation of a replication attempt
Source: PLoS One. 2022 Jan 27;17(1):e0262718. doi: 10.1371/journal.pone.0262718 (PMC8794161; doi:10.1371/journal.pone.0262718)
Supplement: S1 Table — The intensity rating question read: “To which degree were the following sensations present during stimulation?”. The confidence rating question read: “To which degree do you believe this was caused by the stimulation?”. (DOCX) [file pone.0262718.s002.docx]

| Intensity rating^1^ | | | | | | Confidence rating^2^ | | | | |
| --- | --- | --- | --- | --- | --- | --- | --- | --- | --- | --- |
|  | none | a little | moderate | strong | very strong | n/a | unlikely | possibly | likely | very likely |
| **anodal session** | |  |  |  |  |  |  |  |  |  |
| burning | 23 | 11 | 8 | 4 | 0 | 23 | 0 | 2 | 7 | 14 |
| dizziness | 43 | 3 | 0 | 0 | 0 | 43 | 1 | 2 | 0 | 0 |
| fatigue | 22 | 9 | 12 | 2 | 1 | 24 | 13 | 8 | 1 | 0 |
| headache | 32 | 9 | 3 | 1 | 1 | 34 | 1 | 10 | 1 | 0 |
| itching | 18 | 17 | 7 | 4 | 0 | 18 | 1 | 4 | 8 | 15 |
| nauseau | 43 | 2 | 1 | 0 | 0 | 43 | 1 | 1 | 1 | 0 |
| pain | 41 | 3 | 2 | 0 | 0 | 40 | 1 | 0 | 4 | 1 |
| tingling | 11 | 21 | 9 | 5 | 0 | 11 | 0 | 4 | 12 | 19 |
| **cathodal session** | |  |  |  |  |  |  |  |  |  |
| burning | 26 | 9 | 5 | 2 | 1 | 25 | 0 | 1 | 7 | 10 |
| dizziness | 40 | 2 | 0 | 1 | 0 | 39 | 0 | 2 | 1 | 1 |
| fatigue | 15 | 13 | 6 | 8 | 1 | 18 | 12 | 9 | 4 | 0 |
| headache | 30 | 7 | 4 | 2 | 0 | 31 | 0 | 8 | 2 | 2 |
| itching | 18 | 14 | 9 | 2 | 0 | 18 | 0 | 0 | 15 | 10 |
| nauseau | 41 | 2 | 0 | 0 | 0 | 40 | 0 | 2 | 0 | 1 |
| pain | 37 | 4 | 0 | 2 | 0 | 36 | 0 | 1 | 3 | 3 |
| tingling | 5 | 25 | 11 | 2 | 0 | 5 | 0 | 1 | 15 | 22 |

**S1 Table. Number of reports of tDCS side effects.**

^1^ Intensity rating: To which degree were the following sensations present during stimulation?

^2^ Confidence rating: To which degree do you believe this was caused by the stimulation?
