# Supplementary material for: Effects of tDCS on the attentional blink revisited: A statistical evaluation of a replication attempt
Source: PLoS One. 2022 Jan 27;17(1):e0262718. doi: 10.1371/journal.pone.0262718 (PMC8794161; doi:10.1371/journal.pone.0262718)
Supplement: S2 Table — (DOCX) [file pone.0262718.s003.docx]

| Difference | Study 2 | Study 1 | Motivation |
| --- | --- | --- | --- |
| Sample: size | 40 | 34 |  |
| Sample: age | Mean: 20.94, SD: 4.25 | Mean: 22.4, SD: 2.8 |  |
| Sample: gender | 29 female (73%) | 20 female (59%) |  |
| Design: concurrent measurements | EEG | None |  |
| Design: inter-session interval | 1 week | min. 48 hours | Increase similarity between sessions; longer washout time |
| Design: trials per 20-minute block | Short-lag trials: M=130, SD=17; Long-lag trials: M=65; SD=9 | Short-lag trials: 40; Long-lag trials: 40 | Allow as many trials as possible in 20 minutes by making the task self-paced |
| Task: RSVP stream | 15 letters | 17 letters | Shorten the total length of a trial, to allow completion of more trials |
| Task: inter-stimulus interval | 1750 ms, + 1000 ms | 480 ms | Allow EEG responses to return to baseline |
| Task: priming condition | Absent | Present | Remove priming condition as it is orthogonal to main contrast, and showed no effects in @London2021 |
| Task: attentional blink conditions | lag 3, 8 | lag 2, 4, 10 | Increase proportion of trials where T2 was seen, for EEG analysis |
| tDCS: conductive medium | Conductive paste | Saline solution | Prevent bridging EEG electrodes with (spreading) saline solution |

**S2 Table. Differences in the methodology and participant samples of London & Slagter [**[**20**](#ref-London2021)**] (Study 1) and the present study (Study 2).**
